# Early cerebral vasculitic infarcts in acute pneumococcal meningitis

**Published:** 2020-01-05

**Authors:** Kiran Kumar Ramineni, Omprakash Bandaru, Dharmendra Kumar Borad, Ravi Kanth Jakkani

**Affiliations:** 1 Department of Neurology, Yashoda Super Speciality Hospital, Malakpet, Hyderabad, India; 2 Department of Radiology, Yashoda Super Speciality Hospital, Malakpet, Hyderabad, India

**Keywords:** Cerebral Infarct, Pneumococcal Meningitis, Vasculitis

A 20-year-old man without any premorbid illness was brought to the emergency department with the history of high-grade fever and headache since two days. He developed two episodes of generalized tonic clonic seizures and lapsed into altered sensorium. On examination, he was febrile and stuporous with Glasgow coma scale (GCS) score of 10, and had neck stiffness with normal fundus examination.

The possibility of meningoencephalitis was considered. He was intubated, and required mechanical ventilatory support. Haemogram revealed total leucocyte count of 39,500 per microliter with neutrophilic predominance. Other routine laboratory parameters including serology were unremarkable. Electrocardiography (ECG), chest X-ray, and abdomen ultrasound did not reveal any significant abnormality. Computed tomography of brain was normal. Cerebrospinal fluid (CSF) analysis revealed a total count of 1750 cells/cumm with 90% neutrophils, protein of 164 mg/dl, sugar of 24 mg/dl, and adenosine deaminase (ADA) of 2 u/l; Gram staining showed gram-positive cocci in pairs.

Pending the culture reports, he was started on meningitis doses of intravenous ceftriaxone and vancomycin in addition to intravenous levetiracetam and supportive care. By next twenty-four hours, sensorium improved significantly, and was extubated. He was noted to have mild weakness of right upper and lower limbs. 

Magnetic resonance imaging (MRI) of brain with intravenous gadolinium contrast showed leptomeningeal enhancement in bilateral cortical sulci and in basal cisterns (Figure 1, A) and multiple foci of restricted diffusion in subcortical and deep white matter in bilateral cerebral hemispheres more on left side (Figure 1, B). MRA was unremarkable with no evidence of arterial stenosis or occlusion of intracranial arteries (Figure 1, C).

CSF culture confirmed pneumococcal growth sensitive to both ceftriaxone and vancomycin. Work up for alternative causes of stroke in young including vasculitic markers, prothrombotic profile, and trans-esophageal echocardiogram was non-contributory.

**Figure 1:**
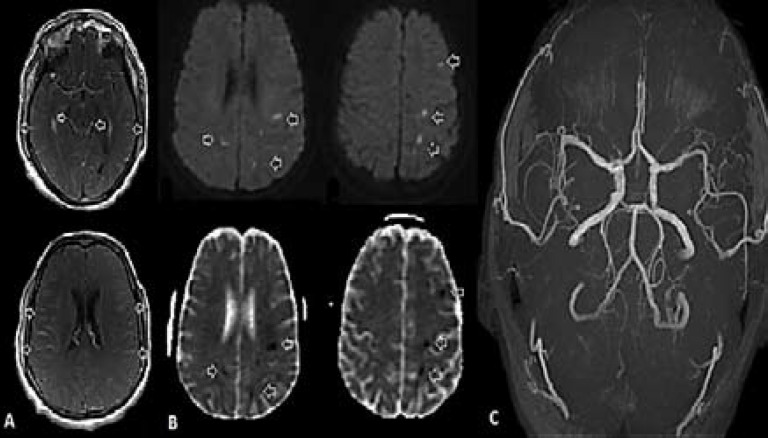
Axial post-contrast magnetic resonance imaging (MRI) images of brain demonstrate leptomeningeal enhancement in bilateral cerebral hemispheres and in basal cisterns (A); axial diffusion-weighted imaging (DWI) images and corresponding axial apparent diffusion coefficient maps demonstrate multiple foci of restricted diffusion in subcortical and deep white matter in bilateral cerebral hemispheres more on left side (B), and axial MRA image demonstrates no evidence of intracranial arterial stenosis/thrombosis (C).

Intravenous dexamethasone (10 mg) four times a day was added and tapered; antibiotics were continued for two weeks. By tenth day, he was asymptomatic without any focal deficits. Final diagnosis of acute pneumococcal meningitis with vasculitic cerebral infarcts was made.

Cerebrovascular complications are known to occur in pneumococcal meningitis.^1^ Ischemic events are more common compared to bleeding. Likely, pathogenesis is inflammation-induced through bacterial blood stream invasion, and activation of the complement and coagulation systems.^2^ The induction of cytokines, oxidative stress, and production of bacterial toxins compromise blood-brain barrier integrity resulting in neurological complications.^3^

Vascular complications usually occur late in the course of illness, and may be preceded by initial clinical improvement.^4^^,^^5^ Our case highlights the rare presentation of pneumococcal meningitis in a young adult with early cerebral vasculitic infarcts. Coupled with meticulous clinical examination, contrast enhanced MRI of brain helps in the timely diagnosis and appropriate management of this relatively uncommon complication in acute bacterial meningitis.

## Notes:

**How to cite this article:** Ramineni KK, Bandaru O, Borad DK, Jakkani RK. Early cerebral vasculitic infarcts in acute pneumococcal meningitis. Curr J Neurol 2020; 19(1): 45-6.
